# An Uncommon Presentation of Ossifying Fibroma in the Maxilla

**DOI:** 10.7759/cureus.23638

**Published:** 2022-03-30

**Authors:** Swetha V Bhat, Santhosh P Kumar, Senthilnathan Periasamy, Vinod K Krishna

**Affiliations:** 1 Oral and Maxillofacial Surgery, Saveetha Dental College and Hospital, Chennai, IND

**Keywords:** cemento-ossifying fibroma, ossifying fibroma, recurrence, benign bone neoplasm, fibro-osseous lesions

## Abstract

Ossifying fibromas are rare, benign, nonaggressive fibro-osseous lesions that manifest in the craniofacial region. Ossifying fibromas are benign tumors of bone, commonly involving the posterior dentate mandible in middle-aged individuals with a female predilection. Clinical manifestations are an asymptomatic expansion of the dentate mandible, with infrequent maxillary lesions. Benign fibro-osseous lesions of the maxilla exhibit similar histopathological findings. Cone-beam computed tomography scan plays an important role in diagnosing and understanding the invasiveness of this lesion. This case report describes an uncommon presentation of ossifying fibroma in the posterior maxilla in an adult male patient. Treatment consisted of surgical excision, and wound healing was uneventful during the one-year postoperative follow-up period.

## Introduction

Fibro-osseous lesions are characterized by a normal bone being replaced by fibrous tissue forming a newly mineralized product. Lesions with osseous and fibrous components include ossifying fibroma (OF), cementifying fibroma, fibrous dysplasia, cemento-ossifying fibroma, and cemento-osseous dysplasia [1]. Fibro-osseous lesions except fibrous dysplasia arise from the periodontal membrane. Ossifying fibroma, a rare tumor entity, is a well-demarcated, benign fibro-osseous monostotic tumor, usually unilocular but occasionally multilocular with a capsule constituted of metaplastic bone, fibrous tissue, and varying amounts of osteoid [2]. OF commonly occurs in middle-aged individuals predominantly in the posterior dentate mandible. OF rarely occurs in the orbit, paranasal sinuses, or the maxillary region and has female predilection [3]. Clinically, these tumors manifest as well-demarcated, slow-growing, asymptomatic intrabony mass and can cause facial deformation due to enlargement over a period of time [4]. The variants of OFs are the conventional and juvenile types. Conventional OF is characteristically slow-growing masses and rarely recurs after treatment. The juvenile type is an aggressive, rapid-growing mass occurring below 15 years of age, radiologically well-demarcated, histologically similar to conventional ossifying fibroma, and recurring in nature [5]. This case report describes an uncommon presentation of ossifying fibroma in the posterior maxilla in an adult male patient.

## Case presentation

**Figure 1:**
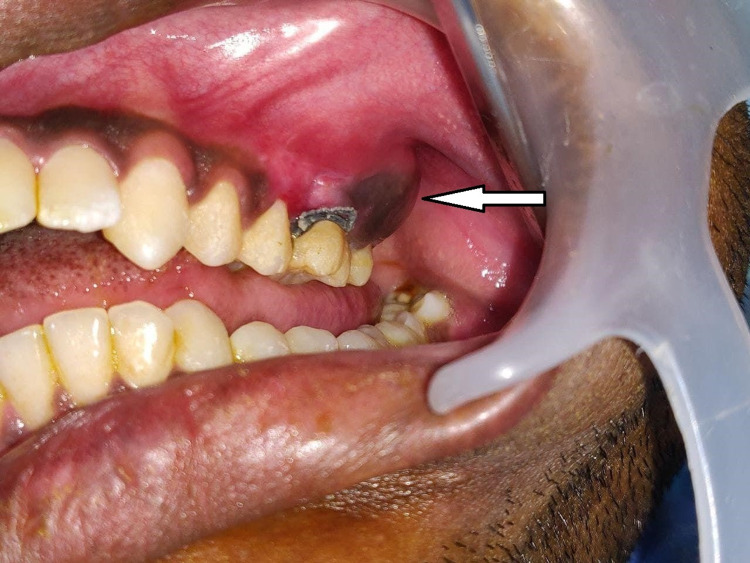
Preoperative intraoral view showing the expansion of the buccal cortex (lesion, arrow) in the left posterior maxillary region

A 22-year-old male patient reported to the Department of Oral and Maxillofacial Surgery complaining of pain and swelling in the left posterior region of his upper jaw for the past six months. The patient had difficulty in mastication due to the presence of swelling. On medical examination, the patient did not have any systemic problems. Extraoral examination revealed facial asymmetry due to the presence of mild swelling on the left middle third of the face. The skin overlying the swelling was normal. Intraoral examination revealed expansion of the maxillary jaw on the left posterior region, extending from the upper left second premolar to the left maxillary tuberosity region, measuring 4.5 × 3.5 cm in size (Figure 1). Buccal expansion of the lesion caused the obliteration of the buccal vestibule with no displacement or mobility of the adjacent teeth. The palatal expansion was not present, and the mucosa covering the lesion on the buccal and palatal sides showed no abnormal changes. No decayed teeth were present in the area of the lesion. Palpation revealed a nontender hard mass with an even surface. Mouth opening was within normal limits, and regional lymph nodes were not palpable.

**Figure 2:**
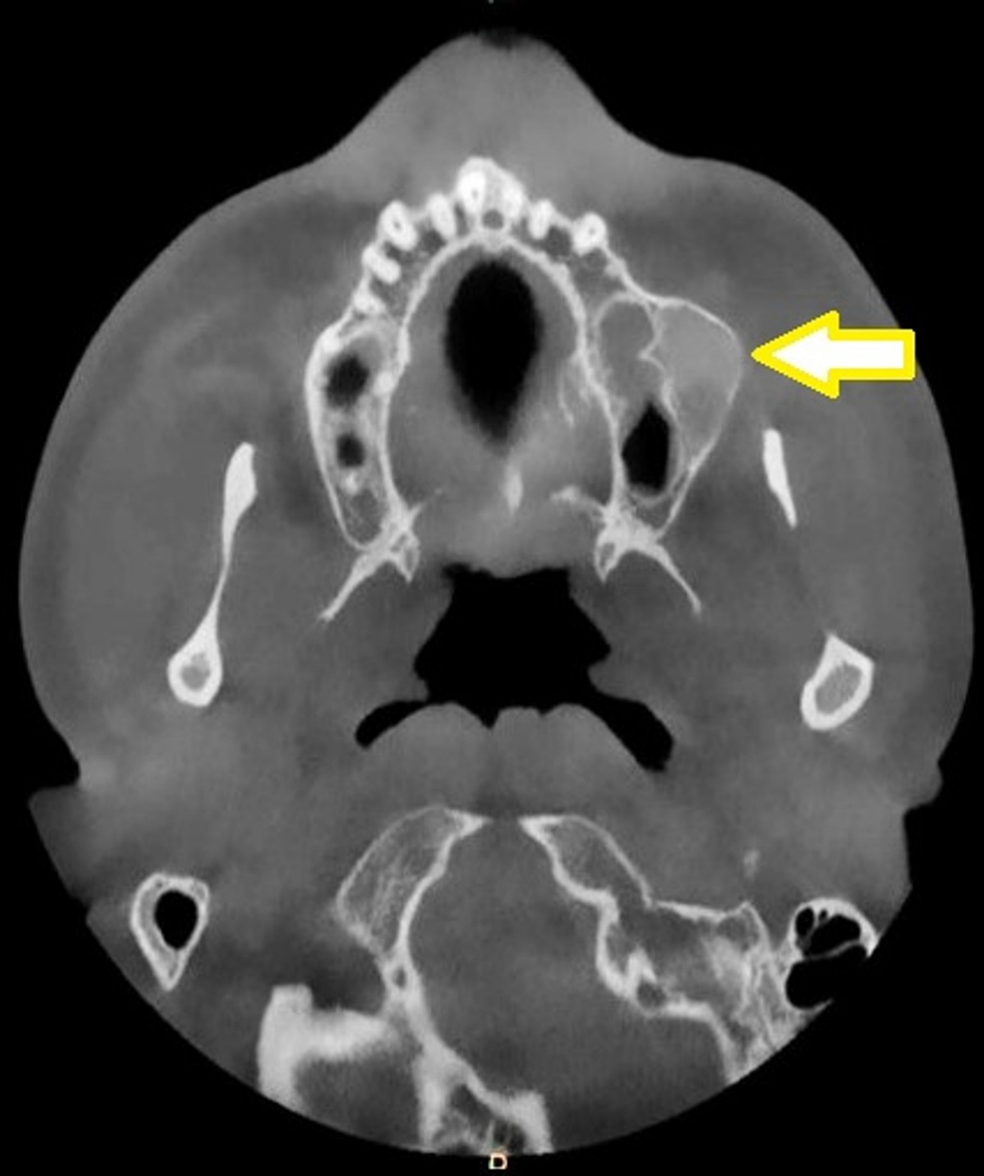
Preoperative CBCT scan (axial section) showing mixed lesion with well-defined borders (arrow) CBCT: cone-beam computed tomography.

**Figure 3:**
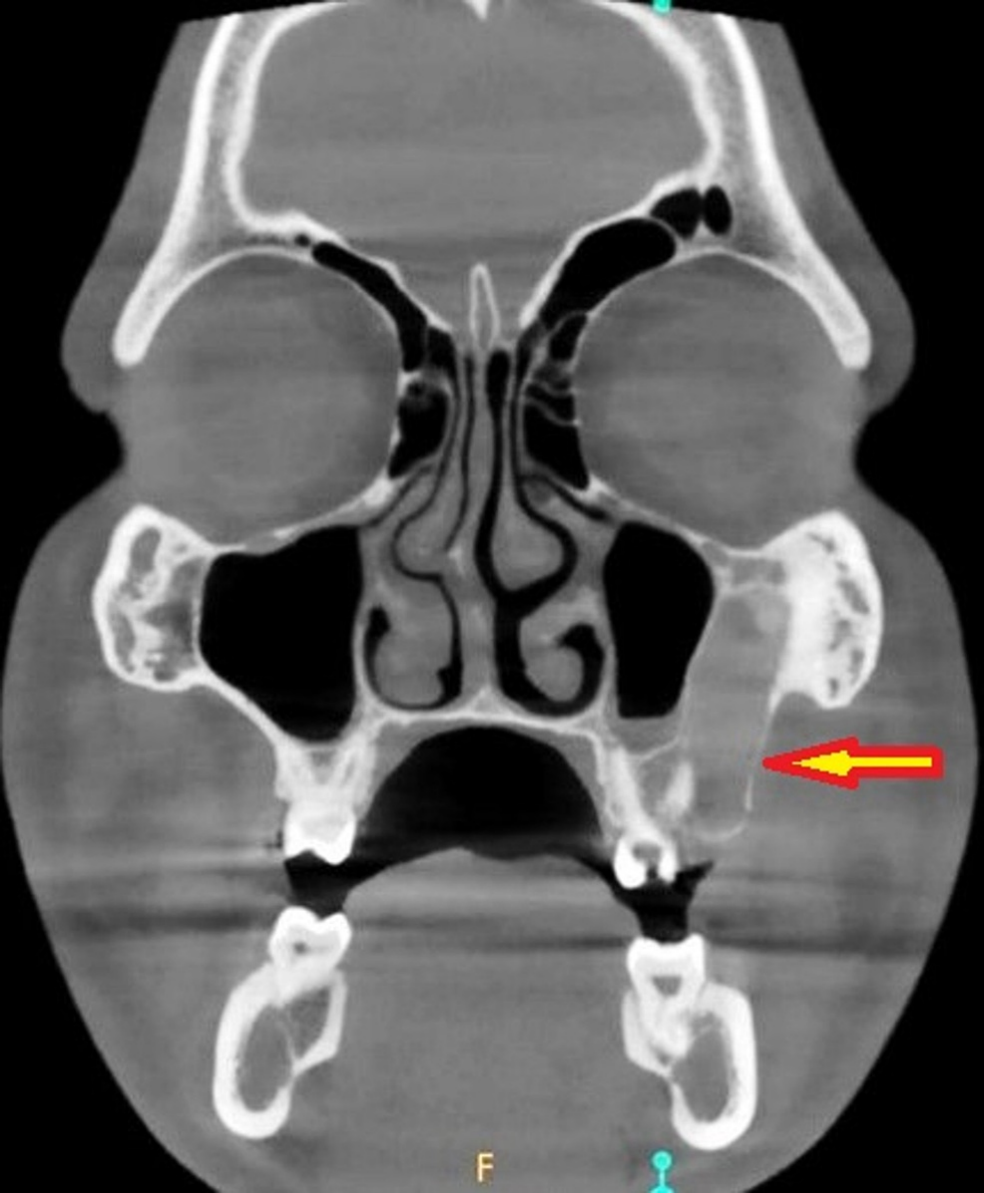
Preoperative CBCT scan (coronal section) showing the invasion of the lesion into the maxillary sinus and the root of the zygoma (arrow) CBCT: cone-beam computed tomography.

The axial section of the cone-beam computed tomography (CBCT) scan revealed a unilocular, mixed (predominantly radiopaque) expansile lesion, with well-defined borders on the left posterior maxilla (Figure 2). The coronal section of the CBCT scan demonstrated the lesion well invading into the maxillary sinus and the root of the zygoma on the left side (Figure 3). The incisional biopsy report suggested the lesion to be ossifying fibroma. Based on the clinical features, history, histological report, and radiological findings, the lesion was diagnosed as ossifying fibroma and planned for surgical excision.

**Figure 4:**
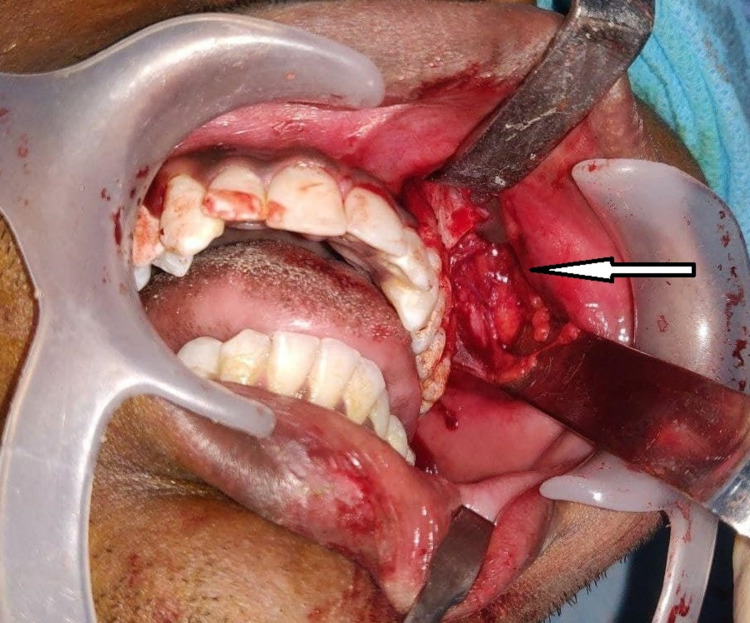
Surgical exposure of the lesion in the left maxilla (arrow)

**Figure 5:**
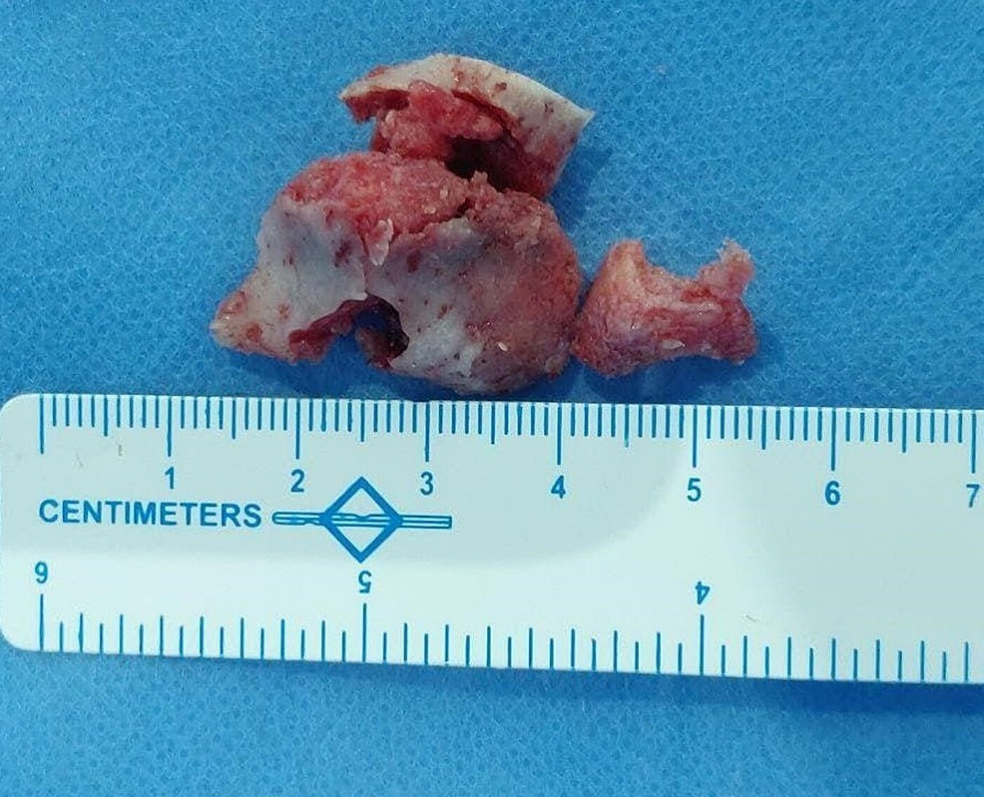
Lesion excised in toto

**Figure 6:**
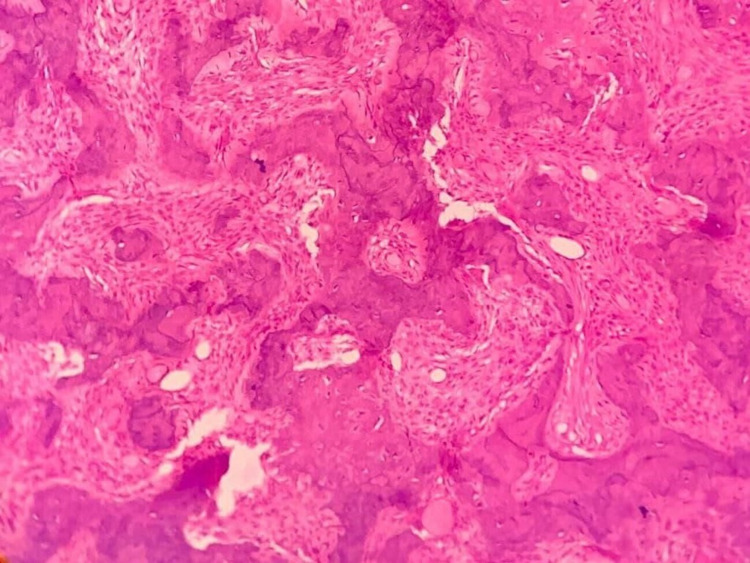
Histology demonstrating features typical of ossifying fibroma

**Figure 7:**
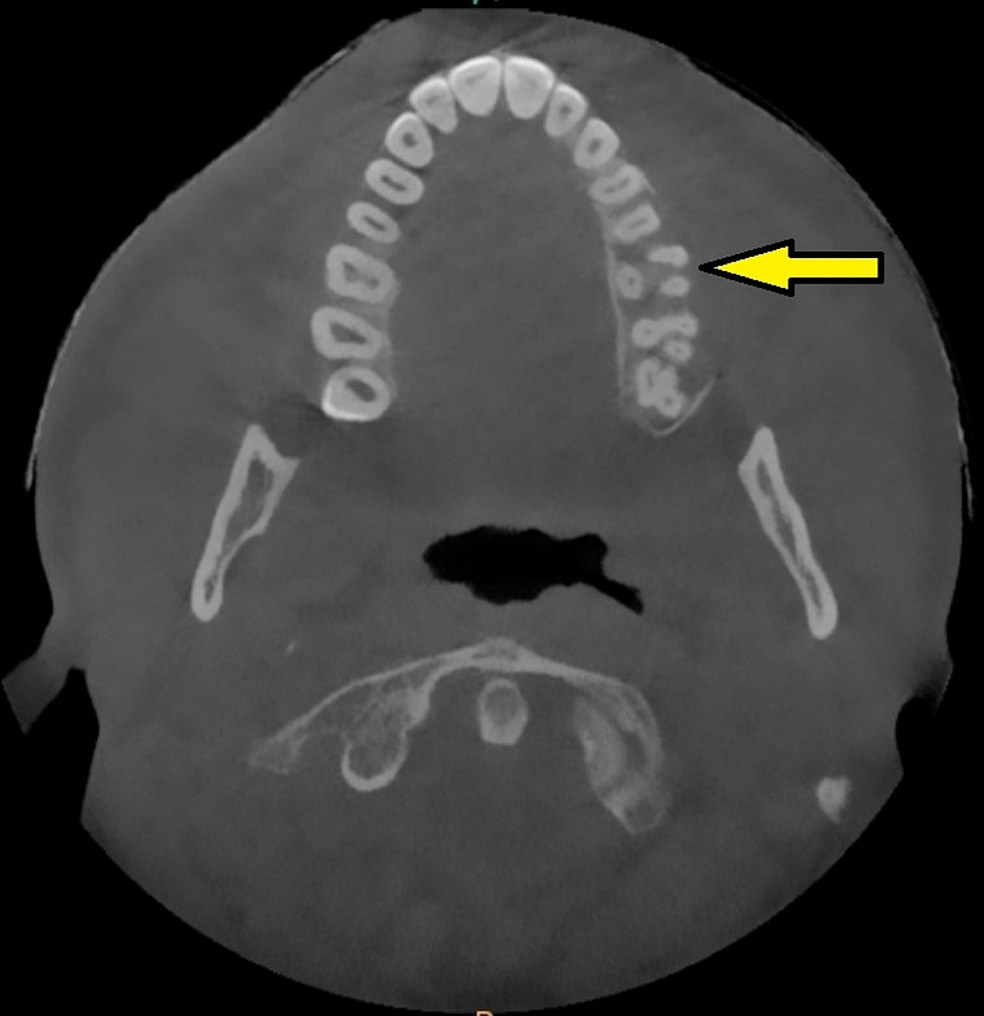
One-year postoperative CBCT scan (axial section, arrow) showing good wound healing CBCT: cone-beam computed tomography.

Under general anesthesia, a vestibular incision was placed in the 22-27 region, the mucoperiosteal flap was raised, and the lesion was exposed (Figure 4). Surgical excision was performed conservatively, and the lesion was removed in toto followed by paring down of the left maxilla and root of the zygomatic process. Excised specimen (Figure 5) was sent for histopathological examination which revealed the presence of several irregular bony trabeculae with osteoid and osteoblastic rimming at the periphery, thus confirming the diagnosis of the ossifying fibroma (Figure 6). An antral pack was placed in the left maxillary sinus, and the wound was closed. One-year postoperative follow-up revealed that the wound healing was uneventful without any signs and symptoms of recurrence (Figure 7).

## Discussion

Although there are differences in classifying and diagnosing fibro-osseous lesions in the craniofacial region, certain clinical features are common to these lesions. These lesions demonstrate the replacement of normal bone tissue with fibroblasts, collagen fibers, with altering amounts of mineralized materials [6]. Pluripotent mesenchymal cells capable of generating cementum, fibrous tissue, and bone give rise to OF. The uncontrolled multiplication of periodontal ligament cells and a metaplastic process arising in connective tissue fibers (nonperiodontal in origin) are the two possible sources of OF, with the former being more common [7]. Ossifying fibroma of the jaws has been linked to a large amount of mesenchymal cellular induction into bone and cementum, which is necessary for odontogenesis. The development of the lesion in the jaws may be steered by a fault in the tissue induction process, trauma, periodontitis, or previous extractions [6].

Ossifying fibroma has a female predilection and occurs predominantly in middle-aged individuals. The mandibular premolar-molar area is the most prevalent site, with only 30% of lesions occurring in the maxilla [3]. Our case exhibited an uncommon presentation in the posterior maxilla in the adult male person. Central ossifying fibromas grow slowly and are usually asymptomatic until they produce any clinically visible expansion [3]. Juvenile aggressive ossifying fibroma, a type of OF occurring between five and 15 years of age, shows more aggressiveness and vascularity [8]. Due to the large fraction of cancellous bone and the sinus present in the maxilla, OFs of the maxilla can expand and grow into enormous sizes [9]. In our case, the lesion was not big and the patient did not present with significant clinical symptoms or any gross conspicuous facial deformation.

Radiography plays an important role in distinguishing OF from other fibro-osseous diseases of the jaw. Only a few lesions are radiolucent, while the vast majority appear as distinctly mixed density lesions (predominantly radiopaque), and it is determined by the maturity of mineralized contents. Few aggressive ossifying fibromas of the maxilla resemble ground-glass appearance [10]. The typical OF grows in a centrifugal pattern, expanding in all directions, and leading to cortical expansion parallel to the growth area.

Histologically, OF is made up of fibrous tissue with varying degrees of mineralized material and cellularity. The bone may have osteoblastic rimming and spherical calcified deposits that are very acellular and resemble cementum. In fibrous dysplasia, the absence of coherent rimming of osteoblasts in the bony trabeculae is utilized to discern it from this lesion, which is more typically lined by plump osteoblasts [11].

The composition of stroma in OF is highly cellular, reflecting the aggressive nature of the tumor, and necessitating thorough surgical excision to prevent local recurrence. Treatment of small lesions involves enucleation and curettage; however, larger lesions need radical resection [12]. Conservative treatment is considered the therapy of choice as it results in minimal morbidity, no loss of sensation as well as no requirement of bone graft, good consolidation, and faster bone formation. Radical resection of the tumor should be considered only in aggressive cases and recurrent lesions due to the destructive nature of this lesion [13]. The reported rates of recurrence range from 14% to 38%, which could be related to inadequate removal or differences in follow-up periods [14,15]. The nature of this lesion demands reviewing the patients for a longer period as there are chances of recurrence, and our patient was symptom free during the one-year postoperative follow-up period.

## Conclusions

The maxillary OF is a very uncommon benign tumor. Because OF is clinically asymptomatic, esthetic and local dental issues are frequently the first signs. CBCT plays a vital role in diagnosing and understanding the invasiveness and extent of this lesion into the adjacent structures. Irrespective of the treatment modality employed, the elimination of the possible etiologic factor is of utmost importance. As OF is a well-circumscribed lesion, the majority of lesions should be treated by surgical excision in a conservative method to minimize facial deformity and loss of function. Radical resection is reserved for aggressive lesions invading adjacent structures. Thorough removal of the lesion is essential to prevent recurrences.
